# Associations of flavonoid intakes with metabolic dysfunction-associated steatotic liver disease in US adults

**DOI:** 10.1371/journal.pone.0322797

**Published:** 2025-05-29

**Authors:** Yuwei He, Yu Chang, Xiangliang Liu, Yuguang Li, Wei Ji, Zhenyu Wang, Jiuwei Cui

**Affiliations:** 1 Cancer Center, The First Hospital of Jilin University, Changchun, China; 2 Breast Surgery Department, The Second Hospital of Jilin university, Changchun, China; Georgia State University, UNITED STATES OF AMERICA

## Abstract

The incidence of Metabolic dysfunction-associated steatotic liver disease (MASLD) is rising annually. Dietary intervention is a cornerstone of MASLD management. Flavonoids, which have anti-inflammatory properties, are thought to benefit MASLD. Using data from the National Health and Nutrition Examination Survey (NHANES) from 2007–2010 and 2017–2018, we conducted a large cross-sectional study. Weighted Logistic regression, Restricted Cubic Spline (RCS) models, and Weighted Quantile Sum (WQS) regressions were used to explore the relationship between Total flavonoid and subclass (Flavones, Flavan-3-ols, Flavonols, Flavonones, Isoflavones, Anthocyanins) intake and MASLD. Participants in the higher tertiles of Total flavonoids intake had 31–34% lower odds of MASLD compared to the lowest tertile intake in the fully adjusted models (Tertile2: OR 0.69, 95%CI 0.55–0.86, P = 0.002, Tertile 3: OR 0.66, 95%CI 0.52–0.84, P < 0.001). Increased intakes of Flavan-3-ols (Tertile 2 in Model 2: OR 0.65, 95%CI 0.49–0.87, P = 0.01), Flavanones (Tertile 3 in Model 2: OR 0.70, 95%CI 0.53–0.91, P = 0.01), and Isoflavones (Tertile 3 in Model 2: OR 0.65, 95%CI 0.52–0.83, P < 0.001) were significantly associated with 30–35% decreased odds of having MASLD. The RCS revealed a significant non-linear dose-response relationship between Total flavonoid, flavonols and MASLD. The WQS model showed that Flavones and Isoflavones had the largest negative contributions to MASLD risk. Our study demontrated a negative correlation between Total flavonoids and their subclasses and risk of MASLD, highlighting the importance of increasing dietary flavonoid intake in the prevention and treatment of MASLD.

## 1. Introduction

Metabolic dysfunction-associated steatotic liver disease (MASLD) has become a major health concern worldwide and its prevalence is increasing globally [1]. In MASLD, which was renamed from non-alcoholic fatty liver disease (NAFLD), the relationship between metabolic dysfunction and hepatic steatosis is more strongly emphasized, including risks such as type 2 diabetes and obesity. The definition of MALD is based on hepatic steatosis and meets at least one of the following three criteria: overweight/obesity, presence of type 2 diabetes, or metabolic dysfunction. This definition is recognized as an adjusted and comprehensive yet concise version. [2,3]. The morbidity and mortality of MASLD are high since it’s closely linked to serious metabolic complications such as obesity, type 2 diabetes and cardiovascular disease [4,5]. It may be related to multiple factors, including abnormal lipid metabolism, oxidative stress and so on [6]. Given the limited therapeutic options for MASLD, it is imperative to develop effective strategies for prevention and treatment.

In recent years, dietary and lifestyle interventions have garnered significant attention for the management of MASLD [7]. Flavonoids, a family of natural polyphenolic compounds widely present in fruits, vegetables, tea, and wine, are considered beneficial for MASLD due to their anti-oxidative and anti-inflammatory properties [8–10]. Previous studies have revealed that higher intakes of Anthocyanins, Flavanones, and Flavonols were inversely associated with metabolic-related diseases, such as MASLD in American adults [11–13]. However, previous studies have not concerned that the role of some subclasses of flavonoids and the combinational effects of flavonoids [14].

Therefore, we performed a cross-sectional study using the continuous National Health and Nutrition Examination Survey (NHANES) to investigate the associations between intakes of Total flavonoids and subclasses (Flavanones, Flavan-3-ols, Flavones, Flavonols, Isoflavones and Anthocyanidins) and the risk of MASLD in US adults. We further applied flexible modeling approaches to explore potential nonlinear relationships and synergistic effects.

## 2. Materials and methods

### 2.1 Study Population

The NHANES is a large-scale cross-sectional survey using stratified and multistage probability sampling methods, aiming to assess the nutritional and health status of adults and children in the United States. The protocol was approved by National Center for Health Statistics (NCHS) ethics review committee, and each participant provided written informed consent (https://www.cdc.gov/nchs/nhanes/index.htm). This is publicly available data and no additional ethical approval or authorization is required.

The survey covers various modules of information including questionnaire interviews, physical examinations, and laboratory tests. The questionnaire interviews are conducted through face-to-face interviews between professionals and participants, while the physical examinations and laboratory tests are collected by professionals on a mobile examination center. These measurements include body measurements such as height, weight, blood pressure, as well as data collection of blood, urine, saliva, etc.

**Fig 1:**
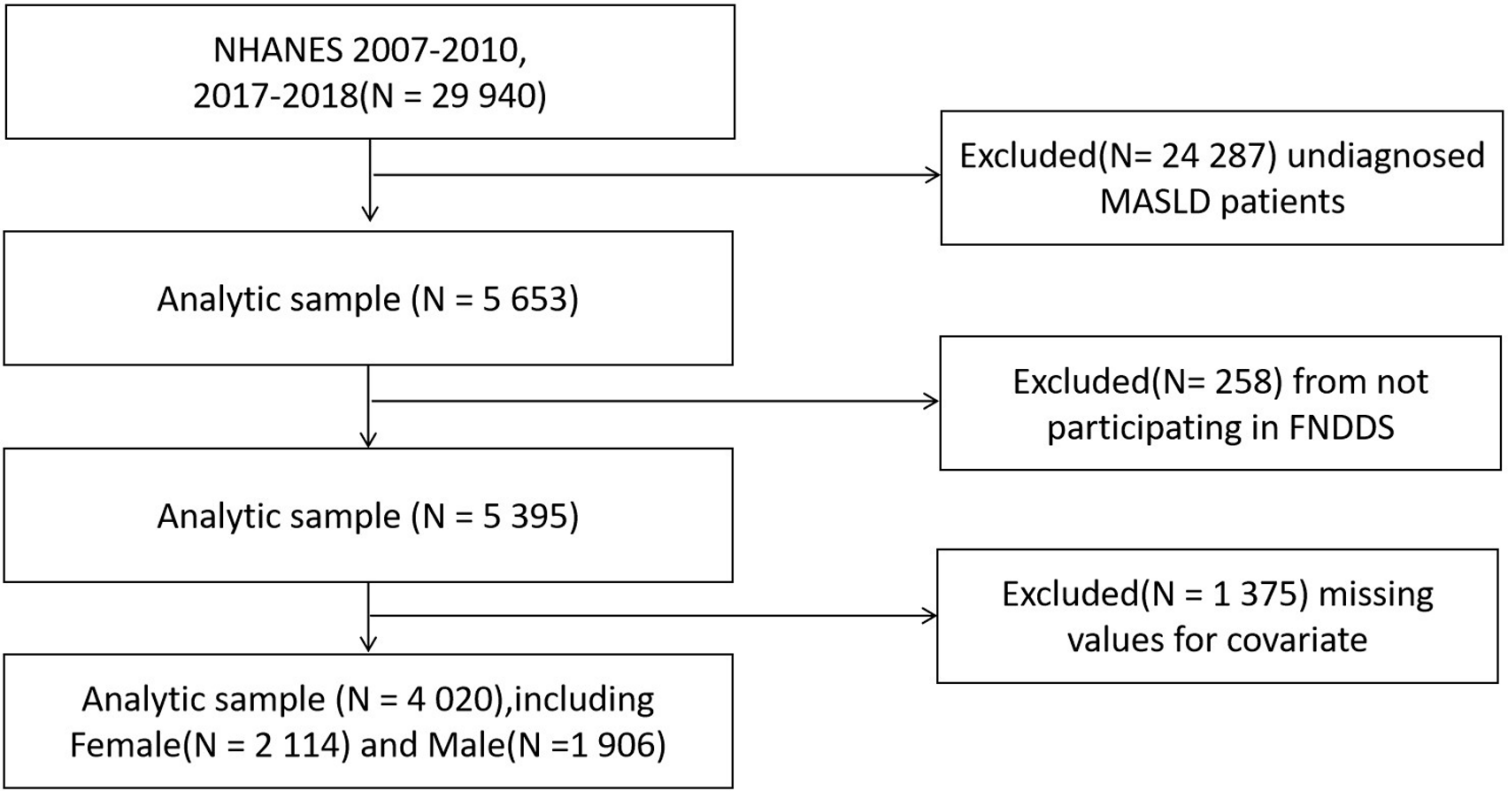
The flowchart of the sample selection from NHANES 2007-2010, 2017-2018.

This study collected data from a total of three cycles (2007–2008, 2009–2010 and 2017–2018), including information from 29,940 participants. We excluded 24,287 participants with missing data that could not be diagnosed as MASLD. Additionally, 258 participants were excluded due to missing information in the Food and Nutrient Database for Dietary Studies (FNDDS) survey conducted by the U.S. Department of Agriculture. 1,374 participants were excluded due to missing data on other covariates. In the end, a total of 4,020 participants were included in this study, with 1,906 male participants and 2,114 female participants. For more details on the screening process, see Fig 1.

### 2.2 Assessment of flavonoid intakes

The intake information of 29 subclasses of flavonoids and 6 subcategories (Flavanones, Flavan-3-ols, Flavones, Flavonols, Isoflavones, Anthocyanidins) was extracted from FNDDS. The daily intake levels of these flavonoid compounds were determined based on the average results of two 24-hour recall questionnaires participated by the participants. The USDA database provides food codes for Total flavonoids and various subclasses of flavonoids that correspond to the NHANES database for the data cycles of 2007–2010 and 2017–2018. This enables further assessment of the daily consumption levels of 29 subclasses of flavonoids and 6 subcategories among the US population. For more details about flavonoids and FNDDS, please visit the following website: https://www.ars.usda.gov/northeast-area/beltsville-md-bhnrc/beltsville-humannutrition-research-center/food-surveys-research-group/docs/fndds-flavonoiddatabase/.

### 2.3 Assessment of metabolic dysfunction-associated steatotic liver disease (MASLD)

Due to the lack of data on liver transient elastography in the NHANES database, we utilized the Fatty Liver Index (FLI) to assess the degree of abnormal liver fat deposition [15]. The construction of this model index was conducted on the general population, based on four clinically accessible indicators: BMI, triglycerides, gamma-glutamyltransferase (GGT), and waist circumference. The specific calculation method is as follows:


$$FLI=\frac{e^{0.953\times ln(triglycerides)+0.139\times BMI+0.718\times ln(gamma-glutamyltransferase)+0.053\times waist\;circumference-15.745}}{1+e^{0.953\times ln(triglycerides)+0.139\times BMI+0.718\times ln(gamma-glutamyltransferase)+0.053\times waist\;circumference-15.745}}\times 100$$

In addition, after excluding other liver diseases related to the aforementioned factors, participants with FLI ≥ 60 were defined as MASLD, and with FLI < 30 could exclude MASLD [15]. Patients with FLI between 30–60 should be validated using Dallas Steatosis Index (DSI) and MAFLD is considered present if DSI > 0 [16]. The specific calculation method of DSI is as follows:


$$\begin{array}{cc} DSI & =-9.4+0.316\;(if\;age\;>50\;and\;female)+\;2.4\;(if\;known\;diabetes)\\ & \;+\;0.02*(equals\;0\;if\;diabetic;\;if\;not\;diabetic\;equals\;the\;glucose\;concentration\;in\;mg/dl)\\ & \;+0.3\;(if\;known\;hypertension)+0.5\;(if\;Hispanic/Asian/Other\;race/ethnicity)+Ln(triglycerides\;in\;mg/dl)\\ & \;+0.4\;(if\;ALT\;13.5-19.49\;IU/L)+1.1\;(if\;ALT\;19.5-40\;IU/L)+1.5\;(if\;ALT\;>\;40\;IU/L)\\ & \;+0.7(if\;not\;black\;and\;BMI\;25-27.49\;kg/m^{2}\;and)+1.4\;(if\;not\;black\;and\;BMI\;27.5-34.9\;kg/m^{2})\\ & \;+\;1.9\;(if\;not\;black\;and\;35-37.49\;kg/m^{2})+\;2.6\;(if\;not\;black\;and\;>\;37.5\;kg/m^{2})\\ & \;-0.2(if\;black\;and\;BMI\;25-27.49\;kg/m^{2}\;and)+0.8\;(if\;black\;and\;BMI\;27.5-34.9\;kg/m^{2})\\ & \;+0.8\;(if\;black\;and\;35-37.49\;kg/m^{2}+1.8(if\;black\;and\;>37.5\;kg/m^{2}) \end{array}$$

### 2.4 Covariates

This study adjusted for potential confounding factors that may influence the association between flavonoid compounds and MASLD, including: Gender (male/female), Age ([18–65), ≥ 65), Race/ethnicity (Mexican American, Non-Hispanic Black, Non-Hispanic White, Other Hispanic, Other Race - Including Multi-Racial), BMI(<25, [25,30), ≥ 30, diabetes states (diabetes(DM), IFG (impaired fasting glucose), IGT (impaired fasting glycaemia), non-diabetes), Smoking status (Former smoker (smoked more than 100 cigarettes in a lifetime, currently non-smoker), “Never smoker” (smoked less than 100 cigarettes in a lifetime, currently non-smoker), “Current smoker” (smoked more than 100 cigarettes in a lifetime, currently smoking some or every day)), Poverty income ratio (PIR) (<1, 1–3, ≥ 3), Alcohol consumption status (Former drinker (previously consumed alcohol but currently abstaining), Never drinker (never consumed any alcoholic beverages), Mild drinker (daily alcohol consumption between 1 and 2 standard drink units), Moderate drinker (daily alcohol consumption not exceeding 4 standard drink units for males and 3 standard drink units for females), Heavy drinker (daily alcohol consumption exceeding 4 standard drink units for males and 3 standard drink units for females)), Education attainment (Low high school, High school, College or above).

### 2.5 Statistical analyses

Considering the complex stratified sampling characteristics of the NHANES database, we followed the guidelines provided by NCHS and conducted weighted analysis using the weights recommended by the NHANES database. Weighting factors included two-day dietary interview weights (WTDR2D), strata (SDMVSTRA), and primary sampling units (SDMVPSU) were considered to account for the complex survey design. Continuous variables and categorical variables were described using weighted means (± standard errors) and sample counts (weighted percentages) respectively. To assess the differences in baseline characteristics between the MASLD and non-MASLD groups, weighted mean differences for continuous variables and weighted percentage differences for categorical variables were examined using t-tests and Rao-Scott χ2 tests, respectively. All statistical tests were two-sided, and p-values <0.05 were considered statistically significant. In this study, participants were categorized into tertiles (Tertile 1, Tertile 2, and Tertile 3) based on their Total flavonoid and flavonoid subclass consumption.

This study constructed multiple weighted logistic models to assess the association between flavonoid intake and MASLD, represented by adjusted odds ratios (ORs) and 95% confidence intervals (CIs). Model 1 was the crude model without any adjustment for variables. Model 2: Adjusted for sex, age, and race/ethnicity. Model 3: Additionally adjusted for BMI, diabetes, education level, poverty income ratio, smoking status, and drinking status based on Model 2. The RCS model was constructed to evaluate the dose-response relationship between flavonoid intake and MASLD. The WQS model was constructed to assess the relationship between combined exposure to Total flavonoids and subclasses and MASLD, as well as the weights of individual flavonoid compounds. The basic weighted index model was as follows:


$$(\mu )=\beta_{o}\;+\beta_{1}\left(\sum_{i=0}^{c}\omega_{i}\varphi_{i}\right)\;+\;z^{\prime }$$

$$(\mu )=\beta_{o}\;+\beta_{1}\left(\sum_{i=0}^{c}\omega_{i}\varphi_{i}\right)\;+\;z^{\prime }$$

$\beta_{o}\;$ represents the intercept, $\beta_{1}$ represents the regression coefficient, g (μ) represents any differentiable link function. c represents the number of flavonoid compounds included in the analysis. $\omega_{i}$ represents the weighted exponent and each exponent ranges from 0 to 1 (0≤$\omega_{i}$≤1), and the total sum of weighted exponents is 1. $z^{\prime }$ and Φ represents the matrix and coefficients of the covariates included as weighted indices. ($\varphi_{i}$ =0,1,2,3) represents the quartiles for each flavonoid compound concentration respectively. $(\sum_{i=1}^{c}{\overset{―}{\omega }}_{i}\varphi_{i}$) is the sum of the weighted quartiles of c components. We assumed a linear function fitted to a Gaussian distribution and randomly assigned the data into a training set (60%) and a validation set (40%). The weights were estimated in the training set [17].

## 3. Results

### 3.1. Baseline characteristics

We divided the participants into MASLD and non-MASLD groups based on the presence of MASLD and summarized and analyzed their baseline characteristics in the two groups. There were more female participants in the non-MASLD group, accounting for approximately 58.0%, while there were more male participants in the MASLD group, accounting for approximately 56.26%. Compared to the non-MASLD group, the MASLD group was more likely to consist of males, aged > 65 years, non-Hispanic white, non-smokers, with a PIR between 1 and 3, and mild alcohol drinkers. Compared to the non-MASLD group, the MASLD group had a lower consumption of Isoflavones, while there were no significant statistical differences in the consumption of Total flavonoids, Flavanones, Flavan-3-ols, Flavones, Flavonols, and Anthocyanidins. More baseline characteristics are shown in Table 1.

**Table 1:** Survey-weighted baseline characteristic of the study population by MASLD.

Variable	Level	No MASLD	MASLD	P-value
Sex (%)				<0.001
	Female	58.00(1.87)	43.74(1.53)	
	Male	42.00(1.87)	56.26(1.53)	
Age				<0.001
	[18, 65)	84.07(0.76)	78.10(1.41)	
	≥65	15.93(0.76)	21.90(1.41)	
Race/ethnicity				0.04
	Mexican American	5.31(0.72)	8.06(1.27)	
	Non-Hispanic Black	10.51(1.01)	9.21(1.17)	
	Non-Hispanic White	71.58(1.66)	72.05(2.42)	
	Other Hispanic	4.84(0.63)	4.20(0.77)	
	Other Race – Including Multi-Racial	7.76(0.81)	6.47(1.15)	
BMI				<0.001
	<25	63.71(1.28)	1.12(0.40)	
	[25,30]	32.39(1.23)	27.08(1.69)	
	≥30	3.90(0.47)	71.79(1.60)	
Smoke (%)				<0.001
	Former	21.28(1.30)	29.06(1.67)	
	Never	60.09(1.28)	60.86(1.70)	
	Now	18.63(1.48)	10.08(1.13)	
Poverty (%)				0.24
	<1	12.88(1.11)	11.01(1.35)	
	[1, 3)	32.96(1.78)	36.56(2.48)	
	≥3	54.16(1.58)	52.43(2.58)	
Alcohol drinkers				<0.001
	Former drinker	9.20(1.02)	20.73(1.86)	
	Heavy drinker	17.18(1.07)	0.00(0.00)	
	Mild drinker	46.22(1.86)	63.68(1.94)	
	Moderate drinker	16.34(1.27)	0.00(0.00)	
	Never drinker	11.07(0.92)	15.59(1.18)	
Diabetes status				<0.001
	DM	6.42(0.67)	25.71(1.66)	
	IFG	6.16(0.69)	15.34(1.21)	
	IGT	5.35(0.54)	9.01(0.97)	
	No	81.35(1.20)	49.50(2.06)	
Hypertension				<0.001
	No	76.94(1.59)	43.92(1.66)	
	Yes	23.06(1.59)	56.08(1.66)	
Education (%)				0.01
	Low high school	11.96(0.80)	17.33(1.37)	
	High school	24.06(1.40)	24.72(1.96)	
	More than high school	63.98(1.82)	57.94(2.53)	
TG(mean (SE))		86.58(1.32)	165.72(5.03)	<0.001
TC(mean (SE))		186.18(1.18)	200.01(1.73)	<0.001
LDL-C(mean (SE))		108.39(0.91)	121.30(1.36)	<0.001
Flavanones(mean (SE))		11.84(0.73)	11.54(1.07)	0.82
Flavan-3-ols(mean (SE))		173.49(12.19)	216.31(18.23)	0.05
Flavones(mean (SE))		1.02(0.07)	0.93(0.05)	0.17
Total ﬂavonoid(mean (SE))		224.17(13.49)	263.66(18.86)	0.07
Anthocyanidins(mean (SE))		15.19(1.11)	13.70(1.79)	0.43
Isoflavones(mean (SE))		2.70(0.45)	1.16(0.29)	0.01
Flavonols(mean (SE))		19.93(0.81)	20.02(0.67)	0.93

BMI, body mass index; DM, diabetes; IFG: impaired fasting glucose; IGT: impaired fasting glycaemia; TG, Triglyceride; TC, Total Cholesterol; LDL-C: Low-Density Lipoprotein Cholesterol; SE: standard error.

### 3.2. Association between flavonoids and six subclasses intake and MASLD

The relationship between flavonoids and intake of six subclasses and MASLD was evaluated using multiple logistic regression models. In model 1, compared to Tertile 1 group of intake, the risk of MASLD was reduced by 31% in Tertile 2 group (OR 0.69, 95%CI 0.55–0.86, P = 0.002). In model 2, compared to Tertile 1 group of intake, the risk of MASLD was reduced by 34% in Tertile 2 group (OR 0.66, 95%CI 0.52–0.84, P < 0.001).

In model 1, compared to Tertile 1 group of Flavan-3-ols intake, the risk of MASLD was reduced by 32% in Tertile 2 group (OR 0.68, 95%CI 0.510.92, P = 0.01). In model 2, compared to Tertile 1 group of Flavan-3-ols intake, the risk of MASLD was reduced by 35% in Tertile 2 group (OR 0.65, 95%CI 0.490.87, P = 0.01), and by 21% in Tertile 3 group (OR 0.79, 95%CI 0.60–1.04, P = 0.09).

In model 2, compared to Tertile 1 group of Flavanones intake, the risk of MASLD was reduced by 30% in Tertile 3 group (OR 0.70, 95%CI 0.530.91, P = 0.01).

In model 1, compared to Tertile 1 group of Isoflavones intake, the risk of MASLD decreased by 32% (OR 0.68, 95% CI 0.54–0.85, P < 0.001). In model 2, compared to Tertile 1 group of Isoflavones intake, the risk of MASLD decreased by 35% (OR 0.65, 95% CI 0.52–0.83, P < 0.001). In model 3, compared to Tertile 1 group of Isoflavones intake, the risk of MASLD decreased by 29% (OR 0.71, 95% CI 0.49–1.04, P = 0.08).

In model 2, compared to Tertile 1 group of Anthocyanidins intake, the risk of MASLD decreased by 32% (OR 0.68, 95% CI 0.31–0.90, P = 0.010). In model 2, compared to Tertile 1 group of Anthocyanidins intake, the risk of MASLD decreased by 33% (OR 0.67, 95% CI 0.46–0.90, P = 0.010). For more details, please refer to Table 2.

**Table 2:** Survey-weighted association of flavonoids and six subclasses intake with MASLD.

Total ﬂavonoid intake									
	Model 1			Model 2			Model 3		
	OR	95% CI	P-value	OR	95% CI	P-value	OR	95% CI	P-value
**Total ﬂavonoid**									
Tertile 1	Reference			Reference			Reference		
Tertile 2	0.69	0.55, 0.86	0.002	0.66	0.52, 0.84	<0.001	0.84	0.56, 1.24	0.36
Tertile 3	0.82	0.64, 1.06	0.12	0.81	0.63, 1.05	0.10	1.22	0.83, 1.80	0.30
**Flavones intake**									
	Model 1			Model 2			Model 3		
	OR	95% CI	P-value	OR	95% CI	P-value	OR	95% CI	P-value
**Flavones**									
Tertile 1	Reference			Reference			Reference		
Tertile 2	0.82	0.63, 1.07	0.13	0.80	0.61, 1.06	0.12	0.76	0.50, 1.18	0.21
Tertile 3	0.89	0.67, 1.16	0.38	0.84	0.63, 1.13	0.25	0.80	0.52, 1.25	0.31
**Flavan_3_ols intake**									
	Model 1			Model 2			Model 3		
	OR	95% CI	P-value	OR	95% CI	P-value	OR	95% CI	P-value
**Flavan_3_ols**									
Tertile 1	Reference			Reference			Reference		
Tertile 2	0.68	0.51, 0.92	0.01	0.65	0.49, 0.87	0.01	1.07	0.66, 1.73	0.78
Tertile 3	0.80	0.60, 1.05	0.10	0.79	0.60, 1.04	0.09	1.08	0.71, 1.66	0.70
**Flavonols intake**									
	Model 1			Model 2			Model 3		
	OR	95% CI	P-value	OR	95% CI	P-value	OR	95% CI	P-value
**Flavonols**									
Tertile 1	Reference			Reference			Reference		
Tertile 2	0.89	0.70, 1.13	0.32	0.86	0.67, 1.09	0.20	0.84	0.55, 1.28	0.39
Tertile 3	0.88	0.71, 1.10	0.25	0.83	0.67, 1.02	0.07	0.94	0.60, 1.45	0.76
**Flavanones intake**									
	Model 1			Model 2			Model 3		
	OR	95% CI	P-value	OR	95% CI	P-value	OR	95% CI	P-value
**Flavanones**									
Tertile 1	Reference			Reference			Reference		
Tertile 2	0.96	0.77, 1.19	0.68	0.98	0.79, 1.23	0.86	1.28	0.84, 1.96	0.24
Tertile 3	0.70	0.54, 0.91	0.01	0.70	0.53, 0.91	0.01	0.98	0.68, 1.40	0.89
**Isoﬂavones intake**									
	Model 1			Model 2			Model 3		
	OR	95% CI	P-value	OR	95% CI	P-value	OR	95% CI	P-value
**Isoﬂavones**									
Tertile 1	Reference			Reference			Reference		
Tertile 2	0.98	0.77, 1.25	0.85	0.99	0.76, 1.28	0.91	0.82	0.56, 1.20	0.29
Tertile 3	0.68	0.54, 0.85	<0.001	0.65	0.52, 0.83	<0.001	0.71	0.49, 1.04	0.08
**Anthocyanidins intake**									
	Model 1			Model 2			Model 3		
	OR	95% CI	P-value	OR	95% CI	P-value	OR	95% CI	P-value
**Anthocyanidin**									
Tertile 1	Reference			Reference			Reference		
Tertile 2	0.82	0.64, 1.05	0.11	0.82	0.63, 1.06	0.12	0.78	0.56, 1.09	0.14
Tertile 3	0.68	0.51, 0.90	0.01	0.67	0.49, 0.90	0.01	0.72	0.44, 1.18	0.18

Model 1: No covariates were adjusted.

Model 2: Adjusted for sex, age and race.

Model 3: Adjusted for sex, age, race, education level, poverty income ratio, BMI, diabetes, smoking status and drinking status.

OR, odd ratios; CI, confidence interval

### 3.3. Nonlinearity analysis using restricted cubic spline (RCS)

**Fig 2:**
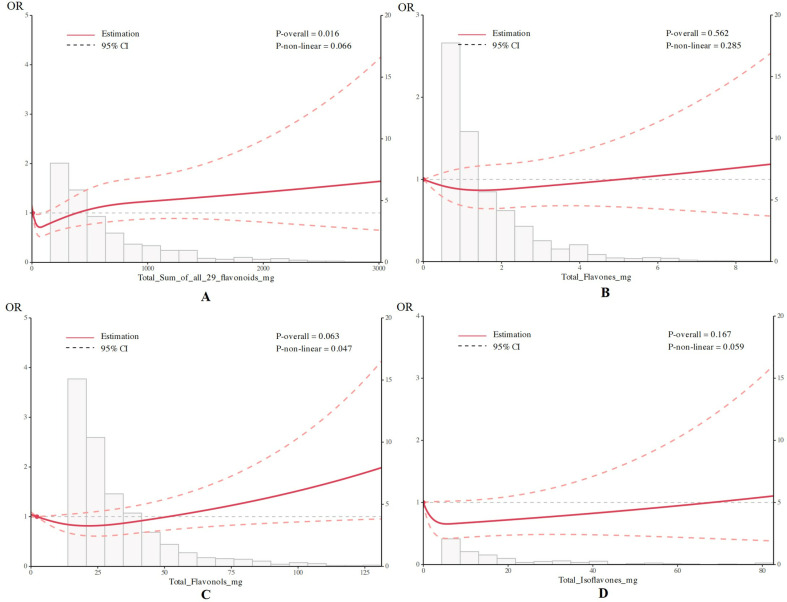
Association between flavonoid intakes and metabolic dysfunction-associated steatotic liver disease (MASLD) using a Restricted Cubic Spline regression model, adjusted for age, sex, race/ethnicity, education level and poverty-income ratio, BMI, diabetes, smoking status and drinking status. (A)Total flavonoids. (B) Flavones. (C) Flavonols. (D) Isoflavones.

The dosage-response relationship between the consumption of flavonoid compounds and MASLD was analyzed using RCS. Nodes were established at the 5th, 35th, 65th, and 95th percentiles of the consumption level for Total flavonoids, Flavones, Flavonols, and Isoflavones, with the 5th percentile serving as the reference value. Adjustments were made for gender, age, race, education, PIR, BMI, diabetes, smoking status, and alcohol consumption. The analysis demonstrated a significant association between the consumption of Total flavonoids and the occurrence of MASLD (p for overall association = 0.016, p for nonlinear association = 0.066) (Fig 2A). No significant differences were observed between the consumption of Flavones and the occurrence of MASLD, and the nonlinear relationship was not significant (p for overall association = 0.562, p for nonlinear association = 0.285) (Fig 2B). The consumption of Flavonols showed a significant nonlinear association with the occurrence of MASLD (p for overall association = 0.06, p for nonlinear association = 0.047) (Fig 2C). No significant differences were observed between the consumption of Isoflavones and the occurrence of MASLD, and the nonlinear relationship was not significant. More details can be seen in Fig 2.

### 3.4. Weighted quantile sum (WQS) regression

**Fig 3:**
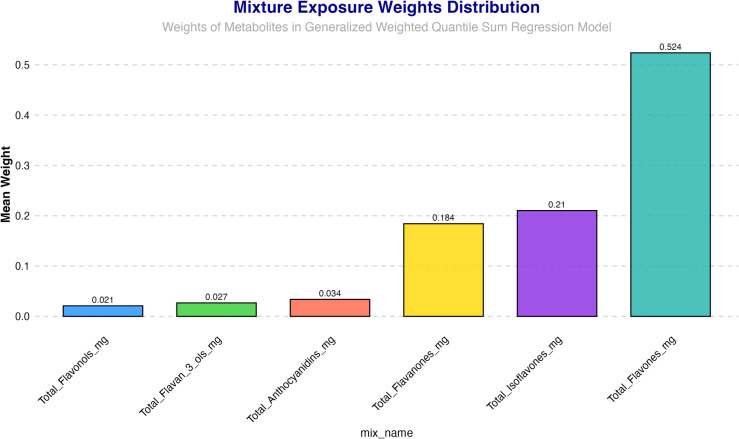
Weighted Quantile Sum model regression index weights for the MASLD. MASLD: metabolic dysfunction-associated steatotic liver disease.

A weighted index was constructed in a WQS model, adjusting for gender, age, race, education, PIR, smoking status, and alcohol consumption, to estimate the cumulative effects and weight distribution of Total flavonoids and six subclasses intake on MASLD. The WQS index showed a positive correlation with the occurrence of MASLD (OR (95%): 0.87 (0.77, 0.98), P = 0.018). Among them, Flavones (52.39%) and Isoflavones (21.03%) had the highest weight proportions, and Flavones (18.42%) and Anthocyanidibs (3.4%) had the secondary higher weight proportion. For more specific details, please refer to Fig 3.

## 4. Discussion

Our study found that Total flavonoids and subclasses intake were inversely associated with MASLD risk. Participants in the higher tertiles of Total flavonoids intake had 31–34% lower odds of MASLD compared to those in the lowest tertile intake in the fully adjusted models [11]. Higher intakes of Flavan-3-ols, Flavanones and Isoflavones were also significantly associated with 30–35% decreased odds of having MASLD. Moreover, we observed linear dose-response relationships between Total flavonoids and MAFLD, and nonlinear dose-response between Flavonols and MASLD, with steeper risk reduction at lower intake levels. In the WQS regression assessing combinational effects, Flavones and Isoflavones contributed the most to the inverse association with MASLD. Our findings added novel evidence that greater dietary flavonoids, especially Flavan-3-ols, Flavanones, Flavones, Anthocyanidins and Isoflavones, may protect against MASLD. The nonlinear and synergistic relationships warrant confirmation in future studies.

The inverse associations between flavonoid subclasses and MASLD risk may be explained by several potential mechanisms. For instance, Flavan-3-ols such as epigallocatechin gallate (EGCG) can alleviate endoplasmic reticulum stress, regulate lipid metabolism related genes, and improve insulin resistance in the liver, thus attenuating steatosis [18]. Flavanones like naringenin and hesperetin can enhance fatty acid oxidation and inhibit de novo lipogenesis by modulating the AMPK/ACC pathway [19].

Isoflavones such as genistein and daidzein may reduce reactive oxygen species, regulate adipocytokines, and modulate gut microbiota to exert anti-inflammatory effects [20]. Anthocyanidins such as cyanidin-3-glucoside can ameliorate dyslipidemia, hyperglycemia and oxidative stress through the PI3K/Akt/GSK-3β pathway [21,22].

Our comprehensive analyses failed to demonstrate a relationship between flavonols and MAFLD, although flavonols have been shown to clear reactive oxygen species and reduce oxidative stress. This may be attributed to their relatively low bioavailability and complex metabolic pathways in vivo, despite their strong physiological activities.[23]

The synergistic effects among flavonoid subclasses are probably mediated through complementary mechanisms like improving insulin sensitivity, preserving gut barrier integrity, and inhibiting hepatic cholesterol synthesis [24]. Our results provided biological plausibility for the observed associations. Further mechanistic studies are warranted to better elucidate the modes of action. Given that MAFLD is a metabolic disorder with numerous risk factors, we adjusted for several covariates in our analysis, including gender, age, demographic characteristics, BMI, diabetes, alcohol consumption history, and smoking history. This approach was intended to minimize the potential influence of these factors on the results, thereby yielding more reliable and scientifically sound conclusions.

Flavonoids and phenolic acids, as natural antioxidants, play a protective role in hepatic steatosis and NASLD [25,26]. At present, the relationship between flavonoids and MASLD has attracted much attention [27], but the flavonoids subclasses and their combined effects on MAFLD have rarely been explored. Our research aimed to systematically examine the associations between six flavonoid subclasses and MASLD risk. We expanded current evidence by including more comprehensive flavonoid subclasses and applying flexible modeling approaches. Specifically, RCS models and WQS models were utilized to explore potential nonlinear relationships and synergistic effects of flavonoids on MASLD [28,29]. Our findings provided novel insights into the protective effects of flavonoids against MASLD. The data-driven modeling enhanced our understanding of the mechanisms underlying flavonoid effects in MAFLD prevention. The results also highlighted the importance of assessing combined influences when studying dietary patterns in relation to complex diseases [30].

The major strengths of this study included using nationally representative NHANES data, which encompassed a large sample size of US adults [31]. The continuous 2-year cycles enhanced statistical power and allowed examination of temporal trends. Additionally, the fatty liver index was calculated as a surrogate marker for MASLD, which improved feasibility and avoided selection bias related to imaging or biopsies [32]. However, it was not clear whether there was steatosis in patients with FLI between 30 and 60[15], so DSI was selected for further screening to ensure the real and accurate population after the screening process[16].

We adopted a comprehensive modeling strategy, and the consistent findings from logistic regression, splines analysed and WQS regression reinforced the reliability of our results [33]. These innovations in study design, surrogate definition and statistical analyses helped ensure robust evidence regarding the beneficial effects of flavonoids on MASLD.

There are several limitations in this study. The cross-sectional design precluded causal inference, and the associations need to be confirmed in prospective cohorts and randomized trials. We did not have access to liver biopsy samples to evaluate pathological changes directly. The self-reported dietary data may be subject to recall biases. Additionally, residual confounding from unmeasured factors could not be excluded.

Going forward, longitudinal studies with repeated dietary assessment are warranted to validate our findings and establish temporal sequences. The collection of biological samples will help elucidate mechanisms and examine metabolite profiles. Future randomized controlled trials testing flavonoid supplementation are needed to determine the interventional effects on MASLD in patients. Our study set the stage for further investigating the preventive and therapeutic potential of flavonoids against MASLD.

## 5. Conclusions

In conclusion, this cross-sectional study found higher intakes of Total flavonoids and subclasses, especially Flavan-3-ols, Flavanones, Anthocyanidins and Isoflavones, were significantly associated with lower MASLD risk in US adults. The nonlinear dose-response and potential synergistic relationships were elucidated through flexible modeling approaches. These results add to the growing body of evidence supporting the favorable effects of flavonoid-rich foods on MASLD. Potential mechanisms may involve enhancing insulin sensitivity, alleviating oxidative stress and modulating lipid metabolism. Further prospective studies and randomized trials are warranted to establish causal relationships and confirm the interventional benefits. Our findings highlight the importance of increasing dietary flavonoid intake for MASLD prevention and treatment.
